# The Fabrication of Nanostructures on Polydimethylsiloxane by Laser Interference Lithography

**DOI:** 10.3390/nano9010073

**Published:** 2019-01-07

**Authors:** Jun Wu, Zhaoxin Geng, Yiyang Xie, Zhiyuan Fan, Yue Su, Chen Xu, Hongda Chen

**Affiliations:** 1 Key Laboratory of Optoelectronics Technology, Ministry of Education, Beijing University of Technology, Beijing 100124, China; wdison@emails.bjut.edu.cn (J.W.); xuchen58@bjut.edu.cn (C.X.); 2 School of Information Engineering, Minzu University of China, Beijing 10083, China; 3 State Key Laboratory of Integrated Optoelectronics, Institute of Semiconductor, Chinese Academy of Sciences, Beijing 100083, China; fanzhiyuan@semi.ac.cn (Z.F.); suyue@semi.ac.cn (Y.S.); hdchen@semi.ac.cn (H.C.)

**Keywords:** laser interference lithograph, PDMS, periodic nanostructures

## Abstract

We report a method for fabricating periodic nanostructures on the surface of polydimethylsiloxane (PDMS) using laser interference lithography. The wave-front splitting method was used for the system, as the period and duty cycle can be easily controlled. Indium tin oxide (ITO) glass reveals favorable characteristics for controlling the standing waves distributed in the vertical direction, and was selected as the rigid substrate for the curing of the PDMS prepolymer, photoresist spin coating, and exposure processes. Periodic nanostructures such as gratings, dot, and hole arrays were prepared. This efficient way of fabricating large area periodic nanoscale patterns will be useful for surface plasmonic resonance and wearable electronics.

## 1. Introduction

The fabrication of nanostructures on flexible substrates has attracted the attention of many researchers in recent years. Flexible materials possess a series of excellent properties, such as transparent, bendable, wearable, and high transmission that the rigid ones do not have. A number of photoelectric and biological devices, for instance, nano-generators [1], sensors [2], and micro-flow paths [3], require flexibility to broaden their applications. Nano-patterns have extensive and prominent applications in various fields, because they are the bases for the realization of different phenomena and functions [4,5,6,7,8]. Therefore, the fabrication of nanoscale patterns on flexible substrates with a low cost, large area, and high throughput using laser interference lithography will have both commercial and scientific interests.

Flexible materials are normally classified into thermoplastics and elastomers [9]. Common materials such as polyethylene terephthalate (PET), polymethyl methacrylate (PMMA), polycarbonate (PC), polystyrene (PS), polyimide (PI), polypropylene (PP), and so on, are good examples of thermoplastics. While polydimethylsiloxane (PDMS), as a kind of silicone-based organic polymer and elastomer material, has plenty of significant advantages over both rigid and thermoplastic materials. The intrinsic chemical structure grants PDMS favorable properties. The organic backbone is responsible for its mechanical elasticity and inert physiochemical characteristics, and siloxane chains provide a high thermal stability [10]. In addition, PDMS has excellent light transmission at both visible and ultraviolet wavelengths. Furthermore, the PDMS prepolymer is prepared by thoroughly mixing the PDMS curing agent with the PDMS base monomer, and then various shapes can be obtained by putting the prepolymer into silicon molds, after the curing process at low temperatures. The silanized Si wafer will facilitate in the following release of the patterned PDMS layer, which can be retransferred to other kinds of substrates by bonding. Consequently, the fabrication of nanostructures on the surface of PDMS provides possibilities for a series of subsequent applications.

For microscale fabrication with highly reproducible procedures, photolithography has been the most reliable and widely used pattern fabrication process in the semiconductor industry. Although traditional mask lithography can be used to fabricate patterns on flexible materials [11], the preparation of structures with nanoscale resolution cannot be realized because of the limitations of the ordinary lithography process. Besides photon lithography technology, writing with particles, such as electron beam lithography (EBL), can also be applied to fabricate flexible nanostructures [12]. The de Broglie wavelengths of the electrons are well below 0.1 nm, allowing for the fabrication of ultrahigh resolution nanostructures [13]. However, this method is evidently limited by being time consuming, low yields, high cost, and small pattern area, which is not suitable for mass production. Furthermore, the instability of the beam, due to drifting, and the inconsistent exposure, due to the fluctuations in the current, tend to occur because of the serial patterning process. Soft lithography, proposed by Whitesides, is another method of fabricating flexible nanostructures [14]. The technique is becoming mature, but the process of making periodic nanostructures is relatively cumbersome and is mainly used in the field of microfluidic chips [15]. Moreover, the flexibility of the elastic material limits its accuracy. The distortion and deformation of the elastic mold may also lead to a great influence on the yields at the same time. Nano-imprint lithography (NIL), as the most commonly used approach in the flexible nanostructures manufacturing field, has the advantages of high resolution and throughput. Numerous identical patterns can be printed continuously with only one template. Nevertheless, this method has to be silanized in order to separate the template (typically etched Si wafer) from the patterned PDMS film, increasing the cost and complexity of the process. Meanwhile, the toxicity of the silanized reagent increases the risk and requires special attention during the silanization procedure. The imprinted nanostructures can no longer be adjusted unless the predecessor template is replaced, which leads to another drawback. The most crucial disadvantage is that thermal stress tends to occur during the heating and cooling processes, breaking the PDMS away from the substrate [16]. The large mechanical force tends to deform the template, resulting in low fidelity structures and an even more fatal break on the template. Therefore, it is necessary to adopt a method to fabricate periodic nanostructures, which is more efficient, low cost, and nondestructive (less damage) to the PDMS surface.

In this paper, a simple and convenient method for preparing periodic nanostructures is introduced. Gratings, dot, and hole arrays were produced on the surface of PDMS films using laser interference lithography with a wave-front splitting system. Compared to the previous means analyzed above, interference lithography has the advantages of not being very time consuming and of having simple processing, high throughput, low-cost, and a large pattern area [17,18,19,20]. The experimental results show that it is a promising method for manufacturing periodic nanostructures on flexible substrates, especially on PDMS.

## 2. Materials and Methods

### 2.1. PDMS Thin Film Preparation

Indium tin oxide (ITO) glass was selected as the rigid substrate in the experiment. First of all, the ITO glass was cleaned using ultra-sonication with acetone, ethanol, and deionized water for 10 min each, and then dried using a nitrogen gas flow. After that, the PDMS prepolymer (Sylgard-184, Dow Corning, Midland, MI, USA) was prepared by thoroughly mixing the PDMS curing agent with the PDMS base monomer (wt:wt = 1:10). The PDMS prepolymer was cured at 80 °C for 1 h using a hot plate after spin-coating on ITO glass to generate PDMS thin films. By adjusting the rotation speed or the viscosity of the PDMS prepolymer, different thicknesses of PDMS films ranging from hundreds of microns down to 15 μm can be obtained. As the PDMS prepolymer has fluidity, the surface needs to be smoothed by standing for 2 h. The advantage of using a hot plate instead of an oven is that the surface of the hot plate is flatter, while an uneven surface will cause a great defect on the interference lithography process, decreasing the size of the uniform pattern produced.

### 2.2. Interference Lithography

SX AR-P 3500/6 (Allresist, GmbH, Strausberg, Germany) was used as the interference lithography photoresist. Dilution is required as the reflected light from the substrate affects the desired pattern, limiting the thickness of the photoresist [21]. The compositions of the diluted photoresist are SX AR-P 3500/6 positive photoresist mixed with AR 300-12 at a ratio of 1:3.5. Before spin coating, an oxygen plasma (110 W, 300 sccm, and 3 min) was adopted to temporarily improve the adhesion of the PDMS surface with the photoresist for the subsequent interference lithography, as the original state of the PDMS surface is hydrophobic. The spin coating process was performed at a speed of 4000 rmp for 27 s, then baked at 100 °C for 2 min. Finally, an approximately 100-nm thick photoresist layer forms on the top of the cured PDMS.

**Figure 1:**
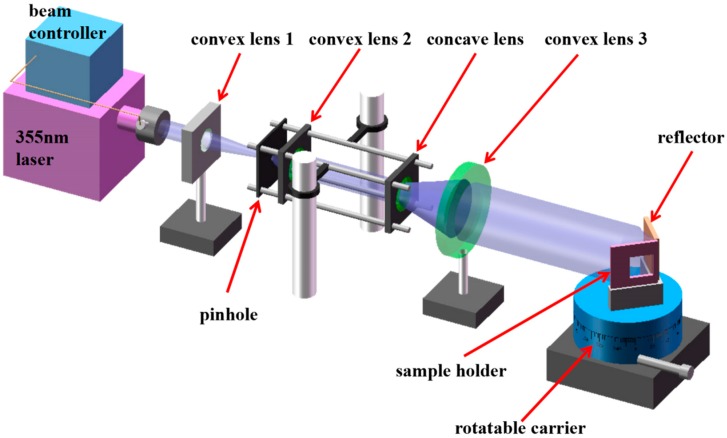
The illustration of interference lithography based on wave-front splitting method for nanostructures fabrication.

**Figure 2:**
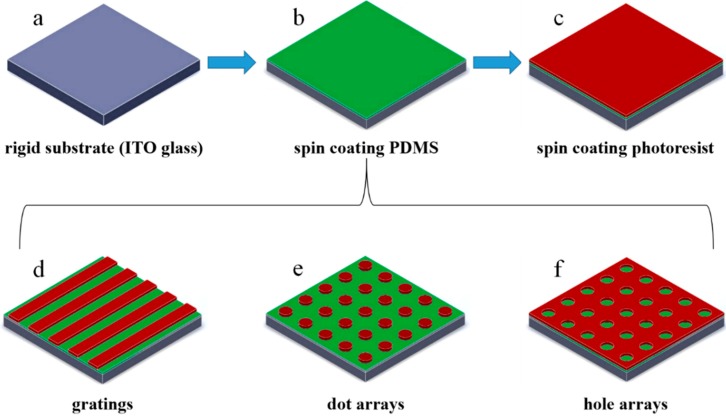
Schematic view of the fabrication process. (**a**) Cleaned ITO glass (**b**) ITO glass coated with a 60 μm thick polydimethylsiloxane (PDMS) preploymer film by spin coating. (**c**) After the curing process, a 100-nm thick photoresist was spin coated on the PDMS surface. (**d**–**f**) The patterns of the nano gratings, dot, and hole arrays fabricated by the interference lithography process.

The schematic of the interference lithography system is illustrated in Figure 1. The optical path is relatively simple when using a wave-front splitting method; besides, the period and duty cycle can be easily controlled. Moreover, the use of fewer mirrors reduced the reflection, as well as the bubbles, scratches, and dust particles attached to the surface. All of these defects will cause a series of secondary wave sources and introduce spatial noise, which ultimately affects the preparation of a large area and of uniform nanostructures. A 355-nm diode-pumped laser (Genesis CX 355, Coherent, Santa Clara, CA, USA) with an output power of 100 mW was selected as the radiation source. This laser has excellent properties, such as a TEM_00_ spatial mode, good beam quality (M^2^ < 1.2), long coherence distance, and relatively compact size (281 × 156 × 85 mm). The radiation was focused usingthe convex lens 1 after passing an optical shutter, then a spatial filter transformed the Gaussian beam into a spherical one. The cage system played collimation and expansion roles, and the convex lens 3 continued to transform divergent into collimated light. At last, the adjusted laser irradiated the sample holder and interfered with the vertical reflector. An optical platform with a damping isolation was used to ensure the stability of the whole system [22]. Optical elements were placed in a closed box to avoid air disturbance and affecting the stability of the interference pattern. The incidence angle can be changed by rotating the rotatable carrier, so that the period of nanostructures is changed accordingly. The fabrication process is shown in Figure 2.

The exposure dose of gratings was about 30 mJ/cm^2^. Although the dot and hole arrays both needed to be exposed twice, with a 90° rotation of the sample holder after the first exposure, the formation of different shapes was determined by the energy distribution. Dot arrays were formed when the duty cycle value was less than 50%, while hole arrays were attained in the opposite way. The doses of the dot and hole arrays were about 15 mJ/cm^2^ and 10 mJ/cm^2^ for every exposure, respectively. According to the exposure dose and the laser power density measured on the PDMS surface, the dosage can be controlled by the optical shutter. After the exposure, AR300-26 (1:20 diluted with deionized water) developer was used to develop the sample for 20 s.

## 3. Results and Discussions

Direct photolithography on flexible substrates have been proven to be a feasible scheme. However, direct interference lithography on a PDMS surface was seldom reported. One of the most important reasons, perhaps, is that the surface topography after the curing process will have a crucial influence on the pattern results, as PDMS is an elastomer material. In addition, PDMS is a transparent material with an excellent transmission within the wavelength range of 300 nm–500 nm [23], so the rigid base on which the PDMS prepolymer is spin coated is also a major factor to be considered. Common rigid substrates, such as silica glass and Si wafer, were used as the supporting substrates for the PDMS prepolymer, but the results are not satisfactory because of their high reflection.

During the interference lithography exposure, two coherent beams with wavelengths of *λ* at an angle of 2*θ* incident the PDMS surface, which is uniformly coated with photoresist. Together with the corresponding substrate reflection components, two standing wave fields are formed in the photoresist layer in horizontal and vertical directions [24,25]. The primary standing waves are parallel to the substrate used to obtain the desired pattern, while the perpendicular one can seriously impact the photoresist profiles if there is not enough suppression. The periodicity (*P_h_*) of the gratings can be simply described as follows:

(1) $$P_{h}=\frac{\lambda }{2\sin\theta },$$

and the period of the vertical standing wave is given by the following:

(2) $$P_{v}=\frac{\lambda }{2n_{p}\cos\theta_{p}},$$

The vertical standing wave period is determined by the following factors: the wavelength of light (*λ*), the refractive index (*n_p_*), and the refracted angle (*θ_p_*) in the photoresist.

**Figure 3:**
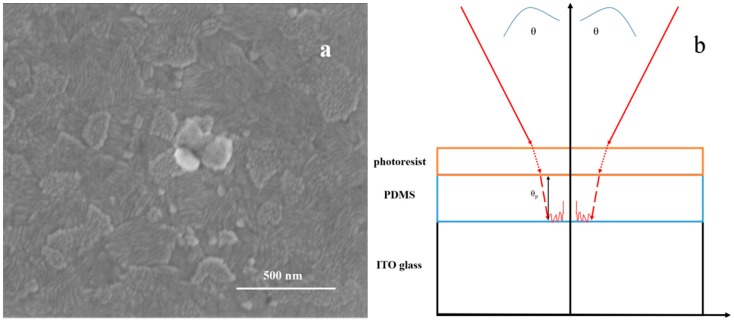
(**a**) Topography of ITO glass under SEM, the pattern showed the surface roughness of the ITO surface. (**b**) Schematic of the wave vectors for double beam laser interference lithography on ITO glass coated with PDMS. Because of the absorption and surface diffuse reflection of transparent ITO glass, the intensity of the beam reflected back to the photoresist is actually very small.

The intensity along the Z-direction is periodic, resulting in uneven distributions. In the region of minimum intensity, the development barrier layer is formed because of an insufficient exposure dose, which will lead to failure. Considering a more extreme case, the development barrier layer can actually cut off the resist structure and separate the top part. Obviously, one effective way to suppress the vertical standing waves is to minimize the reflectivity of the substrate. Anti-reflection coating (ARC) on the substrate surface is a common solution, while ITO glass was introduced in our experiment. As ITO has an absorption in the ultraviolet region [26], for the 355 nm semiconductor laser source we used in the experiment, the ITO’s absorption of ultraviolet rays was in line with the wavelength. Furthermore, as the scanning electron microscope (SEM) image shows in Figure 3a, the surface of the ITO has a relatively rough topography. When the laser passed through the PDMS film, part of it was absorbed by the ITO film, while the other part formed a diffuse reflection on the surface of the ITO glass, so that the laser intensity that reflected back to PDMS was very weak. The standing wave distributed in the vertical direction can be largely ignored accordingly. Therefore, the ITO glass should be the perfect rigid substrate to support PDMS for the laser interference lithography.

**Figure 4:**
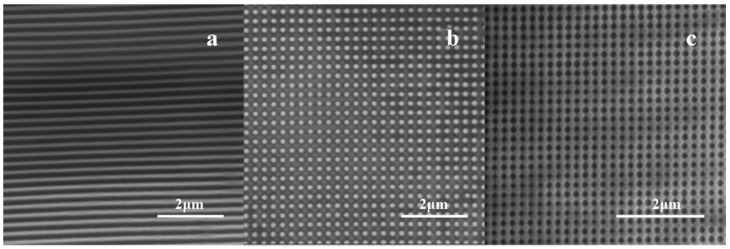
SEM pictures of gratings, dot, and hole arrays of the photoresist on PDMS–ITO substrate. The pictures are magnified by 40,000 times. The period of the grating (**a**) is about 273 nm. The diameter of the dot (**b**) and hole arrays (**c**) is 175 nm and 165 nm, respectively. The dot and hole arrays are made by rotating the sample holder 90° after the first exposure, and the different energy distribution leads to different nanostructures.

**Figure 5:**
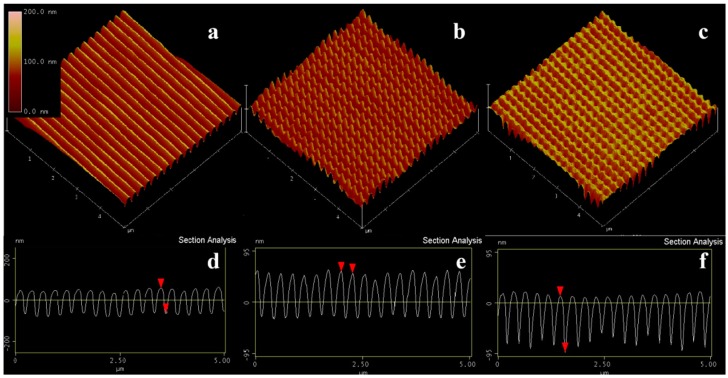
(**a**–**c**) A 25 square microns 3D atomic force microscope (AFM) images of gratings, dot, and hole arrays on PDMS–ITO glass, respectively. (**d**–**f**) The upper nanostructures topology of the photoresist on a PDMS surface.

The SEM images of the PDMS–ITO glass nano-patterns are shown in Figure 4. The sample surface should be sprayed with gold before it can be observed. The photos are magnified by 40,000 times, and the gratings, dot and hole arrays are shown in (a), (b), and (c), respectively. The period of the grating is about 273 nm, which can be modified by rotating the rotatable carrier. The line width is about 100 nm, which is related to the exposure dose and the development time. By adjusting the line width and period, gratings with different duty cycles can be obtained. An area of 25 square microns was also scanned using an atomic force microscope (AFM) (shown in Figure 5), in which the topologies of the photoresist on the PDMS surface are exhibited.

## 4. Conclusions

Three different typical periodic nanostructures were produced, including gratings, dot, and hole arrays. ITO glass was introduced to solve the standing waves distributed in the vertical direction. Compared with the current mainstream nanostructures manufacturing approaches, such as NIL and soft lithography, laser interference lithography is an effective and reliable method that not only has the advantages of having a low cost, high throughput, and being simple to operate, but can also be more convenient to adjust the patterns (period and duty cycle). The combination of flexible materials and nano-patterns can provide applications for localized surface plasmon resonance (LSPR) sensing, photo-voltaic cells, and wearable electronics.

## References

[1] Hinchet R., Seung W., Kim S.-W. (2015). Recent Progress on Flexible Triboelectric Nanogenerators for SelfPowered Electronics. ChemSusChem.

[2] Rim Y.S., Bae S.-H., Chen H., De Marco N., Yang Y. (2016). Recent Progress in Materials and Devices toward Printable and Flexible Sensors. Adv. Mater..

[3] Hill S., Qian W., Chen W., Fu J. (2016). Surface micromachining of polydimethylsiloxane for microfluidics applications. Biomicrofluidics.

[4] Xu K., Chen D., Yang F., Wang Z., Yin L., Wang F., Cheng R., Liu K., Xiong J., Liu Q. (2017). Sub-10 nm Nanopattern Architecture for 2D Material Field-Effect Transistors. Nano Lett..

[5] Ulbricht R., Sakuma H., Imade Y., Otsuka P.H., Tomoda M., Matsuda O., Kim H., Park G.-W., Wright O.B. (2017). Elucidating gigahertz acoustic modulation of extraordinary optical transmission through a two-dimensional array of nano-holes. Appl. Phys. Lett..

[6] Chen W.-Y., Chang H.-Y., Lu J.-K., Huang Y.-C., Harroun S.G., Tseng Y.-T., Li Y.-J., Huang C.-C., Chang H.-T. (2015). Self-Assembly of Antimicrobial Peptides on Gold Nanodots: Against Multidrug-Resistant Bacteria and Wound-Healing Application. Adv. Funct. Mater..

[7] Deng S., Chen R., Zhou W., Ho J.Y.L., Wong M., Kwok H.-S. (2016). Fabrication of High-Performance Bridged-Grain Polycrystalline Silicon TFTs by Laser Interference Lithography. IEEE Trans. Electron Devices.

[8] Seo J.-H., Park J., Zhao D., Yang H., Zhou W., Ju B.-K., Ma Z. (2013). Large-Area Printed Broadband Membrane Reflectors by Laser Interference Lithography. IEEE Photonics J..

[9] Chantiwas R., Park S., Soper S.A., Kim B.C., Takayama S., Sunkara V., Hwang H., Cho Y.-K. (2011). Flexible fabrication and applications of polymer nanochannels and nanoslits. Chem. Soc. Rev..

[10] McDonald J.C., Duffy D.C., Anderson J.R., Chiu D.T., Wu H., Schueller O.J.A., Whitesides G.M. (2000). Fabrication of microfluidic systems in poly(dimethylsiloxane). Electrophoresis.

[11] Diebold R.M., Clarke D.R. (2011). Lithographic patterning on polydimethylsiloxane surfaces using polydimethylglutarimide. Lab Chip.

[12] Nagaraj K.S., Sangeeth K., Hegde G.M., Sirisoonthorn S. (2014). Nanostructure patterning on flexible substrates using electron beam lithography. Proceedings of the International Conference on Experimental Mechanics and Twelfth Asian Conference on Experimental Mechanics.

[13] Qiao W., Huang W., Liu Y., Li X., Chen L.-S., Tang J.-X. (2016). Toward Scalable Flexible Nanomanufacturing for Photonic Structures and Devices. Adv. Mater..

[14] Yin Y., Gates B., Xia Y. (2000). A Soft Lithography Approach to the Fabrication of Nanostructures of Single Crystalline Silicon with Well-Defined Dimensions and Shapes. Adv. Mater..

[15] Bjørnsen G., Roots J., Henriksen L. (2011). Patterning of soft polydimethylsiloxane elastomers using plasma etching. J. Appl. Polym. Sci..

[16] Lan H., Ding Y., Liu H., Que Y., Tao W., Li H., Lu B. (2009). Mold deformation in soft UV-nanoimprint lithography. Sci. China Ser. E Technol. Sci..

[17] Santos A., Deen M.J., Marsal L.F. (2015). Low-cost fabrication technologies for nanostructures: State-of-the-art and potential. Nanotechnology.

[18] Zhang X., Liu H., Feng S. (2009). Solution-processible fabrication of large-area patterned and unpatterned gold nanostructures. Nanotechnology.

[19] Abid M.I., Wang L., Chen Q.-D., Wang X.-W., Juodkazis S., Sun H.-B. (2017). Angle-multiplexed optical printing of biomimetic hierarchical 3D textures: Angle-multiplexed optical printing. Laser Photonics Rev..

[20] Kondo T., Yamasaki K., Juodkazis S., Matsuo S., Mizeikis V., Misawa H. (2004). Three-dimensional microfabrication by femtosecond pulses in dielectrics. Thin Solid Films.

[21] Fang Y., Dai L., Yang F., Yue G., Zuo P., Chen H. (2016). Fabrication of metal nano-wires by laser interference lithography using a tri-layer resist process. Opt. Quantum Electron..

[22] Lin T.H., Yang Y.-K., Mai H.-Y., Fu C.-C., Sanchez M.I., Ukraintsev V.A. (2017). Improved multi-beam laser interference lithography system by vibration analysis model. Proceedings of the SPIE Advanced Lithography.

[23] Zahid A., Dai B., Hong R., Zhang D. (2017). Optical properties study of silicone polymer PDMS substrate surfaces modified by plasma treatment. Mater. Res. Express.

[24] Siddique R.H., Hünig R., Faisal A., Lemmer U., Hölscher H. (2015). Fabrication of hierarchical photonic nanostructures inspired by Morpho butterflies utilizing laser interference lithography. Opt. Mater. Express.

[25] Lin T.-H., Yang Y.-K., Fu C.-C. (2017). Integration of multiple theories for the simulation of laser interference lithography processes. Nanotechnology.

[26] Biyikli N., Kimukin I., Butun B., Aytur O., Ozbay E. (2004). ITO-Schottky Photodiodes for High-Performance Detection in the UV–IR Spectrum. IEEE J. Sel. Top. Quantum Electron..

